# Auditory fear conditioning alters neural gain in the cochlear nucleus: a wireless neural recording study in freely behaving rats

**DOI:** 10.1042/NS20200009

**Published:** 2020-11-16

**Authors:** Antonio G. Paolini, Simeon J. Morgan, Jee Hyun Kim

**Affiliations:** 1 ISN Psychology, Institute for Social Neuroscience, Ivanhoe 3087, Australia; 2 School of Psychology and Public Health, La Trobe University, Bundoora 3086, Australia; 3 Florey Department of Neuroscience and Mental Health, University of Melbourne, Parkville 3052, Australia; 4 IMPACT – The Institute for Mental and Physical Health and Clinical Translation, School of Medicine, Deakin University, Geelong, Australia

**Keywords:** classical conditioning, cochlear nucleus, electrophysiology, learning and memory, neuroplasticity, rat

## Abstract

Anxiety disorders involve distorted perception of the world including increased saliency of stress-associated cues. However, plasticity in the initial sensory regions of the brain following a fearful experience has never been examined. The cochlear nucleus (CN) is the first station in the central auditory system, with heterogeneous collections of neurons that not only project to but also receive projections from cortico-limbic regions, suggesting a potential for experience-dependent plasticity. Using wireless neural recordings in freely behaving rats, we demonstrate for the first time that neural gain in the CN is significantly altered by fear conditioning to auditory sequences. Specifically, the ventral subnuclei significantly increased firing rate to the conditioned tone sequence, while the dorsal subnuclei significantly decreased firing rate during the conditioning session overall. These findings suggest subregion-specific changes in the balance of inhibition and excitation in the CN as a result of conditioning experience. Heart rate was measured as the conditioned response (CR), which showed that while pre-conditioned stimulus (CS) responding did not change across baseline and conditioning sessions, significant changes in heart rate were observed to the tone sequence followed by shock. Heart-rate findings support acquisition of conditioned fear. Taken together, the present study presents first evidence for potential experience-dependent changes in auditory perception that involve novel plasticity within the first site of processing auditory information in the brain.

## Introduction

The ability to modulate attention to biologically significant stimuli is critical for survival. Such a stimulus may be intrinsically salient, or may be initially neutral but become salient due to its association with important events. Pavlovian fear conditioning represents the latter case in which an initially neutral stimulus such as a tone becomes important after repeated pairings with an aversive stimulus, such as a footshock. Fear conditioning has been extensively utilized in humans and rodents to study the potential role of emotional associative memory in anxiety disorders and post-traumatic stress disorder [1–7]. Importantly, while it has been proposed as a useful tool to understand hypervigilance toward cues associated with stressful and/or anxious experience [3,4], how conditioned fear experience may contribute to vigilance at the neural level is poorly understood.

Acquisition of conditioned fear requires the lateral amygdala (LA), which is the locus of convergence of sensory information involved in associative learning [4,8,9]. In the case of tone-fear conditioning, the LA receives auditory information via two well-characterized parallel pathways both of which involve the medial geniculate nucleus of the thalamus (MGN). One pathway consists of the auditory cortex areas that receive input from the MGN and that project to the LA, forming the thalamic-cortico-amygdala pathway [10]. The second pathway involves direct projections from the MGN to the LA, forming the thalamic-amygdala pathway [11,12]. Although conditioning-induced plasticity in the LA has been extensively demonstrated [13–20], whether initial sensory systems also exhibit similar plasticity is unknown. Such plasticity would be important for survival of an organism in its ability to select and process the potentially changing salience of different stimuli in the environment.

The cochlear nucleus (CN) is the first station in the central auditory system [21,22] that project to the amygdala via MGN [23,24]. The CN is heterogeneous collections of neurons broadly divided into dorsal and ventral subnuclei (DCN and VCN, respectively). DCN and VCN are spatially organized to respond to different frequencies of auditory stimuli in rats [25]. For example, each subnuclei show dorsoventral tonotopic organization, with dorsal cells responding to higher frequencies and ventral cells responding to lower frequencies [25–27]. Some tonotopic organization has also been reported mediolaterally [26]. Within DCN and VCN, distinct cell populations are intricately linked. DCN vertical cells inhibit bushy and stellate cells in the VCN [28]. Conversely, inhibitory D stellate cells in the VCN project to vertical cells in DCN and can inhibit their actions [29].

D stellate cells are excited by periolivary olivocochlear bundle activation [30]. The olivocochlear bundle has been implicated in attention [31] suggesting that the tuning properties of VCN cells are not static and can change as a result of the saliency of acoustic input. Interestingly, CN receives projections from the amygdala via nucleus accumbens that modulates MGN [32,33]. Specifically, the olivocochlear bundle receives higher order feedback control indirectly from the LA via its connections to the nucleus accumbens that projects to the MGN [34], which in turn projects to the inferior colliculus [32] providing an avenue for periolivary innervation [35]. This circuitry provides potential top-down processing from the LA to the CN, which may allow experience-dependent plasticity in this initial sensory system.

**Figure 1:**
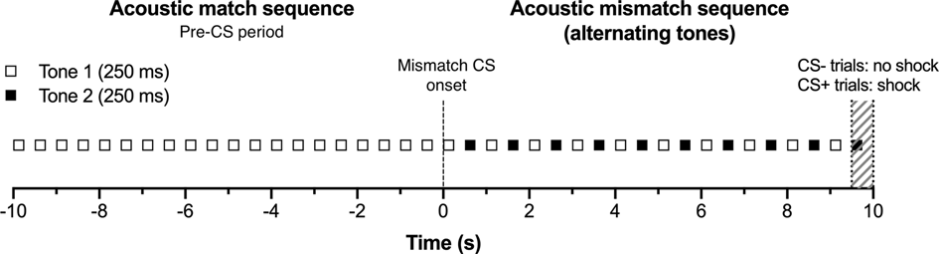
Each acoustic trial consisted of a matched sequence (pre-CS period) immediately followed by a mismatch sequence (CS period) Each match sequence consisted of a period of 80–170 s (only 10-s period shown as an example) during which an initial stimulus (Tone 1) was repeatedly presented. This was followed by the conditioned stimulus (CS) mismatch sequence consisting of a 10-s period in which the initial tone (Tone 1) alternated with a differing tone (Tone 2). Each trial was co-terminated with a footshock in the conditioning (CS+) session while no footshock was presented during baseline (CS−) session.

Therefore, we investigated neural activity in the CN following auditory fear conditioning using wireless electrophysiological recordings in freely behaving rats. Importantly, we developed a unique conditioned tone discrimination paradigm for the present study, in order to capture neural activity in the CN, which is tonotopically organized. Specifically, each trial began with tone pips of a single frequency lasting 80–170 s (Figure 1 shows an example of the last 10 s), immediately followed by a 10-s period in which the initial tone pip alternated with a second tone pip of a different frequency (Figure 1). This tone mismatch sequence served as the conditioned stimulus (CS), whereas the preceding tone match sequence served as a pre-CS control period. Twelve pairs of tones of different frequencies were utilized for the tone mismatch sequence, in order to manipulate the difficulty level of discrimination. That is, a pair of tones that differ by one octave would be more difficult to discriminate from the pre-CS period compared with a pair of tones that differ by four octaves. During the baseline recording session, each trial was not paired with footshock (CS− trials). During the conditioning session, each trial co-terminated with 0.6 mA footshock (CS+ trials). Notably, the shock was only 0.5 s in duration, to ensure minimal electrical interference with the neural recordings. This unique paradigm allowed us to test the CN’s discrimination of the pre-CS vs CS before and after CS-footshock pairings, to ultimately measure its neural gain associated with auditory fear learning.

## Materials and methods

### Animals

Six adult male hooded Wistar rats (Animal Resources Centre, Perth, Australia) were used in the present study, which took place in Paolini Laboratory at La Trobe University. Behavioral data were obtained on all six animals with neural data recorded in four, due to insufficient neural recordings in two. Animals were housed individually on a 12:12-h reverse light–dark cycle (lights on: 7 a.m.) in a temperature-controlled environment, with *ad libitum* access to food and water. All behavioral testing took place during the animals’ dark cycle. Animals were treated according to the principles in The Australian Code of Practice for the Care and Use of Animals for Scientific Purposes (7th ed., 2004. Canberra, Australia), and the ethics application was approved by the La Trobe University Animal Ethics Committee (AEC 09-28P).

### Surgery

The surgery procedures have been previously described [36]. In brief, animals were anesthetized by intraperitoneal injection of ketamine (70 mg/kg) and xylazine (10 mg/kg), and maintained using isoflurane (1-3% vol/vol in oxygen). The electrocardiogram radio telemetry device (TR40; Telemetry Research, TX, U.S.A.) was implanted into the peritoneal cavity, in order to measure heart-rate changes as the conditioned response (CR). Specifically, midline incisions were made extending from the xiphoid process caudally in the skin, the linea alba and the peritoneum, and the device was inserted into the peritoneal cavity. An incision was made in the skin overlying the trachea, exposing the sternohyoid muscle. The positive lead was fed through a subcutaneous tunnel formed between the rostral and caudal incisions, was inserted dorsally into the anterior mediastinum adjacent to the right atrium, then was sutured in place. The xiphoid process was exposed through the caudal incision, and the tip of the negative lead was sutured to the dorsal wall. Excess cable was fed into the peritoneal cavity. The peritoneum and muscle wall were sutured, and skin incisions were closed using Michel Clips.

**Figure 2:**
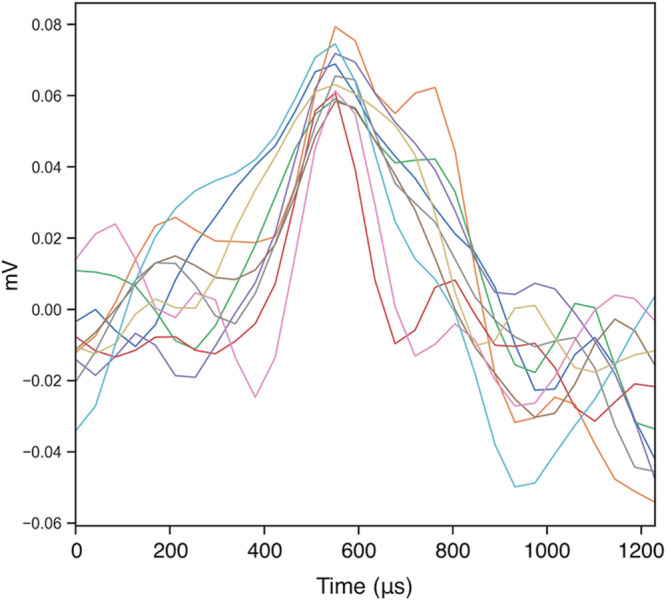
An example of raw multiunit traces in millivolts (mV) from a single channel at the end of electrode implantation surgery when 15 kHz tone was presented for frequency mapping (time at x-axis refers to microseconds from tone onset)

Two weeks after telemetry device implantation, the animals were similarly anesthetized for neural electrode implantation. Head was fixed within a stereotaxic frame (David Kopf Instruments, CA, U.S.A.). Extracellular multichannel electrodes (consisting of two parallel silicon substrate shanks, with 16 iridium electrode sites per shank, 10-mm shanks, 500 μm apart, 413 μm^2^, 100-μm channel intervals, iridium activated, Neuronexus Technology, MI, U.S.A.) were stereotaxically aligned through the cerebellum and into the CN. Once the skin was sutured, frequency response mapping (1–44 kHz, 10–70 dB sound pressure level, 50 ms tone duration) was used to confirm tuned acoustic responses and proving a guide to electrode placement based on broad response profile (Figure 2).

### Conditioning paradigm

Baseline and conditioning sessions were performed in an aluminium-walled test chamber (ENV-009; Med Associates, VT, U.S.A.) with floor area 30.5 cm × 39.4 cm. Footshocks were delivered using an aversive stimulus generator (ENV-410B; Med Associates) and grid scrambler (Solid State Scrambler; Med Associates) through a stainless steel grid floor. The mean background noise level was 34 dB distributed evenly across frequencies. All tones were generated with an RX6 multifunction processor (Tucker-Davis Technologies, FL, U.S.A.) using a programmable attenuator (PA5, Tucker-Davis Technologies) to adjust signal amplitude. Tones were presented through a ceiling-mounted free-field electrostatic speaker (ES1, Tucker-Davis Technologies). All hardware was controlled by custom-built software developed using the OpenEx software platform (Tucker-Davis Technologies). Timing of stimulus presentation and footshock delivery was controlled by the MED-PC IV package (SOF-735; Med Associates) running on a SmartCtrl module (DIG-716B; Med Associates). Custom-built connectors provided interdevice communication.

In order to exploit the tonotopic characteristics of CN, we developed a unique conditioned tone discrimination paradigm involving a single baseline session and a single conditioning session, given 2 days apart. Baseline and conditioning recording sessions were identical, except that no footshocks were present during the baseline recording session. Each session consisted of 48 trials across 1 h and 48 min. Figure 1 provides an abbreviated example of a trial. Each trial lasted 80–170 s (random duration) during which a tone was repeatedly presented (250-ms stimulus followed by 250-ms interstimulus interval; two presentations per second; Figure 1 ‘acoustic match sequence’ pre-CS period), followed by a 10-s period in which the initial tone alternated with a second tone of a different frequency (250-ms initial stimulus, 250-ms interstimulus interval, 250-ms second stimulus, 250-ms interstimulus interval; Figure 1 ‘acoustic mismatch sequence’ CS period). During the baseline session, this mismatch sequence served as a control for the conditioned stimulus (CS−). In the conditioning session, this mismatch sequence served as the conditioned stimulus (CS+), co-terminating with the unconditioned stimulus (US), a 0.5-s footshock (0.6 mA).

For each session, 12 pairs of different pure tone stimuli were used, with each pair presented four times in consecutive trials. Tones covered a range of frequencies (1–44 kHz). Each of the two stimuli served as the initial tone in two trials, and the differing tone in two trials. These four trials were presented in a random sequence. This counterbalancing ensured that the tone added during the mismatch sequence was not disproportionally of higher or lower frequency than the initial tone. Differing tones were 1–5 octaves apart in frequency. For statistical analyses, trials were differentiated into same or lower than 3 (≤3) or higher than 3 (>3) octaves.

### Electrophysiological and heart-rate recordings

All calibration and noise level measurements were conducted using a 1/8 in. pressure-field microphone (4138-A-015; Brüel & Kjær; Nærum, Denmark) and preamplifier (4-Channel Microphone Power Supply, Type 2829; Brüel & Kjær). A Tucker-Davis Technologies RX6 multifunction processor (typical signal-to-noise ratio of 105 dB, sample rate 100 kHz) was used for calibration signal generation and sound level processing.

Before baseline and conditioning sessions, animals were briefly anesthetized using isoflurane (1–3% vol/vol in oxygen for less than 5 min), placed in the test chamber, and a 31 channel wireless multiunit recording headstage (Triangle BioSystems Inc) was fitted. Trials commenced 2 min after normal mobility was observed (∼15 min after the end of anesthesia) as reported previously [31]. A multichannel pre-amplifier (PZ2-256; Tucker-Davis Technologies) sampling at 24414 Hz captured neural activity, which was subsequently band-pass filtered from 300 to 5000 Hz with 24 dB roll-off. Electrocardiogram measured by implanted telemetry device (described above) was digitized at sample rate 2000 Hz using a PowerLab (ML880, ADInstruments, Sydney, Australia) and analyzed using the electrocardiogram analysis module (MLS360/6, AD Instruments). Data were analyzed using the LabChart Pro Electrocardiogram Analysis module (MLS360/6; ADInstruments). To analyze change in heart rate over time in response to CS presentation, the mean proportional change in heart rate during the period from 8 s pre- to 8 s post-mismatch onset normalized to mismatch onset was generated. This was to avoid the shock period in the analyses.

### Histology

**Figure 3:**
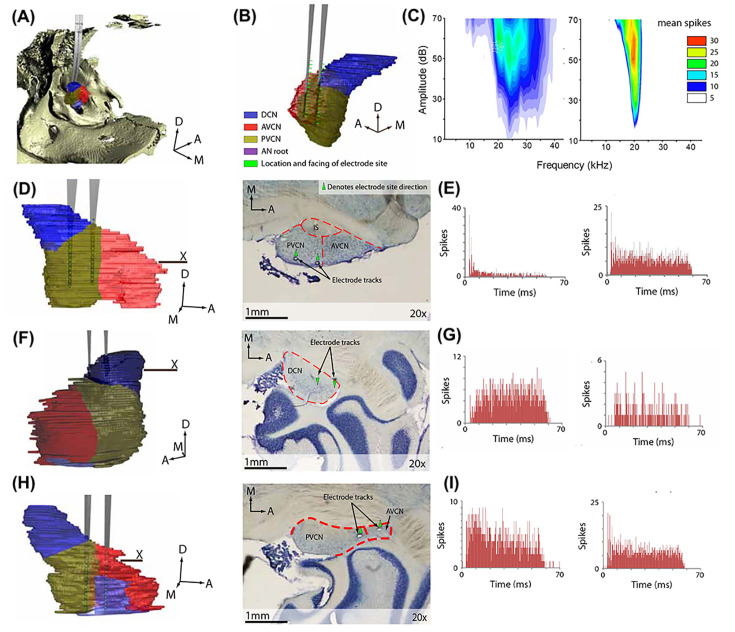
Examples of locations of the implanted electrodes and post-implantation response profile (**A**) A low-power 3D image of CN and surrounding bones (ivory shading) constructed from CT slices, showing electrode position relative to bone and (**B**) CN (blue, dark yellow, pink to reflect dorsal (DCN), anteroventral (AVCN) and posteroventral (PVCN) subnuclei, respectively). (**C**) Typical frequency response areas of neurons near electrode sites. The electrode penetrated the lateral edge of the PVCN and ventral DCN (**D**, left). Electrode tracks are visible in the PVCN in the Nissl-stained section (**D**, right) taken from the horizontal plane marked X. (**E**) Example peristimulus time histograms displaying chopping like multiunit activity (0.1 ms bins, 10 repetitions). Location and typical response profile of recorded multiunits in other animals (**F**–**I**). The profile characteristics of buildup responses, as found in the DCN were evident (**F,G**). Heterogeneous primary and chopper responses typical of VCN (**H, I**). Bin width for peristimulus time histograms is 0.1 ms.

Upon completion of testing, animals were anesthetized by Lethabarb overdose (sodium pentobarbitone, 200 mg/kg i.p.) and transcardially perfused with 10% (vol/vol) formalin in phosphate buffered saline (PBS, pH 7.4). To identify electrode location within the skull, high definition X-ray scans were performed, and computed tomography software (Xradia Inc., U.S.A.) was used to generate slices of 20.6-µm resolution from projections. Three-dimensional reconstructions of the skull and electrode were generated from computed tomography (CT) slices using 3D-Doctor (Able Software, MA, U.S.A.). After scanning, the skull was removed and the brain was sectioned in 50-µm slices using a cryostat (Leica CM 1850, Leica Microsystems, Wetzlar, Germany). Examples of electrode placements are shown in Figure 3A–C,F,H.

## Results

**Figure 4:**
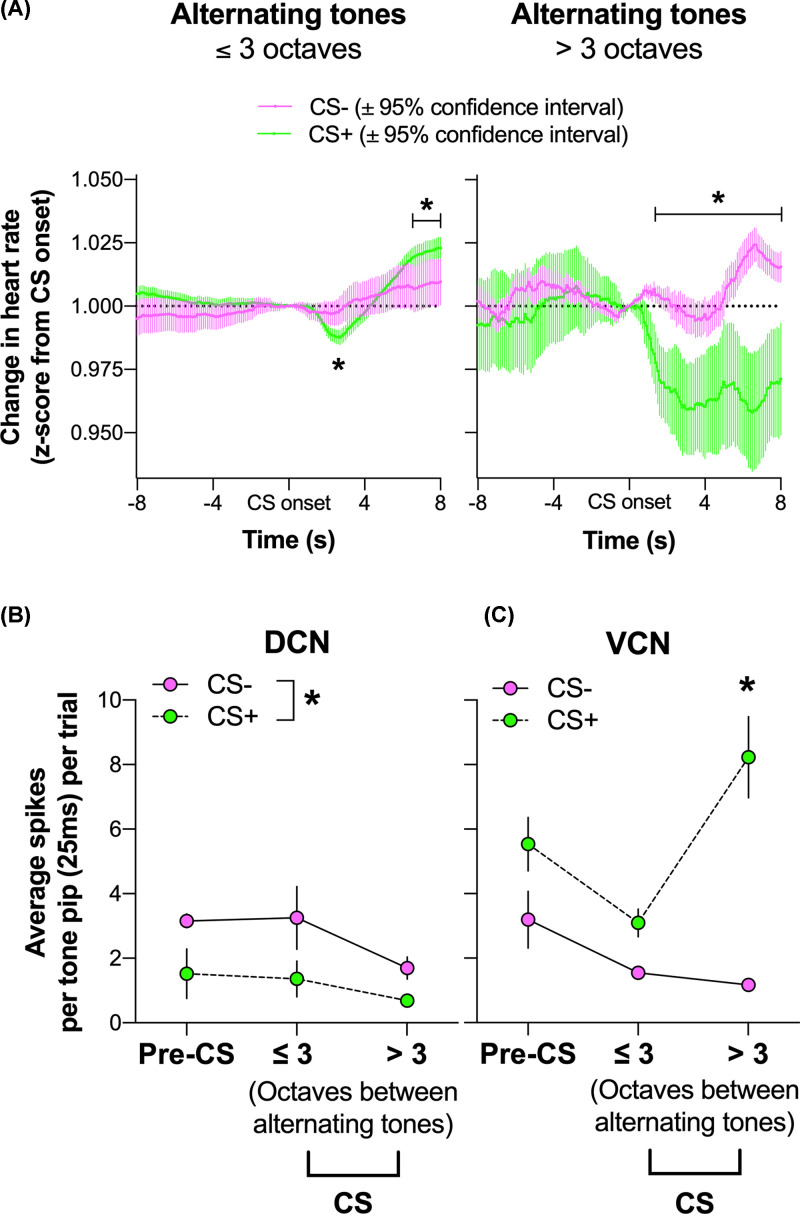
The effect of fear conditioning on heart rate and the neural responsiveness of the CN subdivisions For all panels, responses to CS− and CS+ are represented by the colors pink and green, respectively. (**A**) Proportional change in heart rate as a function of time from mismatch sequence onset (t = 0s) during CS− (pink 95% confidence intervals) and CS+ (green 95% confidence intervals). Responding to pairs of tones were separately plotted based on octave differences between the tones in a CS. For the mismatch sequence under 3 octaves (left), CS+ trials resulted in significantly decreased and then increased heart rate (bradycardia then tachycardia) compared CS− trials during 6.5–8.0 s from mismatch CS onset (*Ps<0.05). CS− and CS+ did not differ outside this interval (Ps>0.05). For the mismatch CS sequence over 3 octaves (right), CS+ trials resulted in significantly decreased heart rate (bradycardia) compared CS− during 1.3–8.0 s from mismatch onset (*Ps<0.05). CS− and CS+ did not differ outside this interval (Ps>0.05), indicating no heart rate changes during pre-CS period. Mean spikes per 250-ms tone pip (± SEM) as a function of different octaves between alternating tones or pre-CS tone, recorded in the (**B**) DCN and (**C**) VCN during CS− and CS+ sessions. In DCN, there were significant main effect of Session (*P<0.05), but no other effects. In VCN, there were significant effects of Session, Type, as well as an interaction between those factors (*P*<0.005). Interaction in VCN was driven by significantly increased firing rate for CS+ compared with CS− for CS with alterrnating tones >3 octaves different in frequency (*P<0.001). Firing rates for CS+ and CS− were similar during pre-CS period and for CSs with tones that differed ≤3 octaves.

Differing tones were 1–5 octaves apart in frequency. For statistical analyses, trials were differentiated into same or lower than 3 (≤3) or higher than 3 (>3) octaves, which were 24 trials each per session. Heart-rate data from six animals were normalized to mismatch CS trial (alternating tones) onset, binned in 100 ms for 8 s before and after the CS onset (Figure 4). For the alternating tones that differed ≤3 octaves, analysis of variance (ANOVA) revealed significant effects of Session [F(1, 480) = 72.62, *P*<0.0001], trial Bin [F(160, 76800) = 8.14, *P*<0.0001], and Session x trial Bin interaction [F(160, 76800) = 2.62, *P*<0.0001]. Interaction was investigated with post hoc tests controlling for family-wise error rate (FWER; due to the huge number of bins = 161), which showed that CS+ tones (green 95% confidence intervals) initially resulted in significantly *decreased* heart rate at 2.7–2.9 s from CS onset and then *increased* heart rate (tachycardia) compared CS− tones (pink 95% confidence intervals) during 6.5–8.0 s from mismatch onset (Figure 4A, *Ps<0.05). CS− and CS+ did not differ outside this interval (Ps>0.05). For the alternating tones that differed more than 3 octaves, ANOVA revealed significant effects of Session [F(1, 94) = 516.00, *P*<0.0001], trial Bin [F(160, 15040) = 2.41, *P*<0.0001], and Session x trial Bin interaction [F(160, 15040) = 3.36, *P*<0.0001]. Post hoc tests controlling for FWER showed that CS+ trials (green 95% confidence intervals) resulted in significantly *decreased* heart rate (bradycardia) compared CS− trials (pink 95% confidence intervals) during 1.3–8.0 s mismatch onset (Figure 4A, *Ps<0.05). CS− and CS+ did not differ outside this interval (Ps>0.05) showing that heart rate to pre-CS tones did not differ between sessions. No effects of Session during pre-CS tones highlight that conditioning occurred specifically to mismatch tones, rather than a general change in heart rate during the conditioning session.

Successful non-isolated multiunit recordings were obtained in four of the six animals from 65 electrode sites. An example of the raw traces is shown in Figure 2. Electrode site characteristic frequencies were identified by review of frequency-amplitude response maps. Electrode placements were confirmed to reside in the posterior VCN (Figure 3A,B,D), DCN (Figure 3A,F) and in the anterior VCN/posterior VCN confines (Figure 3H). Electrode tracks are visible in the posterior VCN and ventral aspects of the DCN in the Nissl-stained horizontal section in (Figure 3D, right). Immediately following implantation (i.e., at the end of surgery), a spatially diverse tonotopic dorsoventral gradient was observed determined by recording frequency amplitude multiunit response maps (shown as power spectrum in Figure 3C). The profile of peristimulus time histograms for numerous sites on electrodes were consistent with multiunit activity typically observed in the area showing chopping/primary like multiunit responses characteristic of neurons of the VCN (Figure 3E,I; 0 ms = onset of tone pip) and build-up responses in the DCN (Figure 3G; 0 ms = onset of tone pip). Recording from multiple regions within one animal was possible and depended on location of recording sites (Figure 3D,H). All four animals’ neural response profiles were indicative of multiunit recordings from the VCN (*n*=44 electrode sites) or DCN (*n*=21 electrode sites) and have been analyzed as such without single-unit sorting, as reported in previous studies [37,38].

Neurons in CN showed subregion-specific plasticity to distinct alternating frequencies of tone paired with shock (Figure 4B,C). Firing rate (spikes per 250 ms tone pip) averaged during 8 s post-mismatch onset was analyzed. A three-way ANOVA that examined differences between recording Area (DCN vs VCN), Session (CS- vs CS+) and tone Type (Pre-CS vs > 3 vs ≤3 octaves) revealed a significant Area × Session × Type interaction [*F (2,378)* = 3.778, *P*<0.0001]. The three-way interaction led to separate analyses of DCN and VCN.

**Figure 5:**
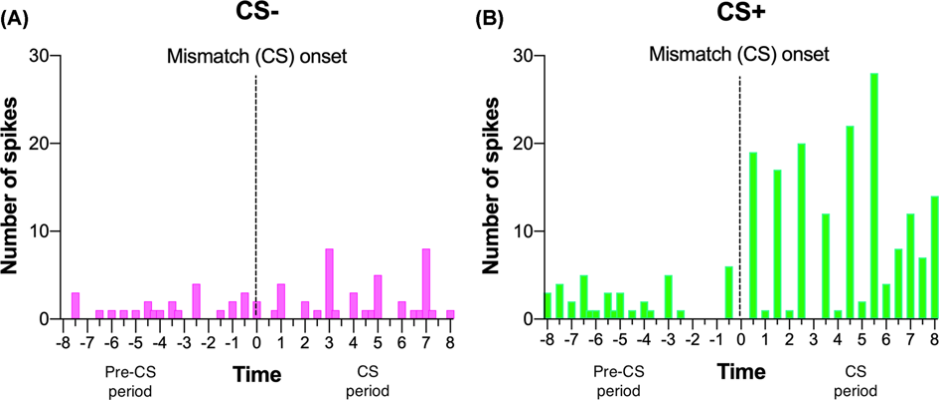
Peri-event histograms of pre-CS and CS responding from an example single electrode site in the ventral cochlear nucleus Each tick on x-axes represent a single tone pip presentation, with between ticks showing inter-pip-interval spikes. (**A**) Recording during the CS− session show minimal changes between pre-CS period and CS− period while recording during the (**B**) CS+ session show significant elevation from pre-CS period to CS+ period. Note minimal responding during inter-pip-intervals.

In DCN, there was only a significant effect of Session [*F (1,120)* = 10.22; Figure 4B, *P<0.05] but no other effects. These findings indicate that regardless of tones presented, conditioning session suppressed firing rate in DCN. That is, the change in DCN response was not learning-specific and was reduced even during pre-CS period. In VCN, however, there were significant effects of Session [*F (1,258)* = 105.6, *P*<0.0001] and Type [*F (2,258)* = 6.128, *P*<0.005], as well as Session × Type interaction [*F (2,258)* = 8.177, *P*<0.0005]. Interaction was followed up with post hoc *t* tests with Bonferroni corrections, which showed that in VCN, firing rate for CS+ is significantly increased compared with CS− for alternating tones >3 octaves different in frequency (Figure 4C, *P<0.001), while CS+ and CS− sessions did not differ for pre-CS period and for CS trials ≤3 octaves (Ps>0.05). This selective increase in VCN firing only for CS+ comprising highly distinctive tones (>3 octaves apart) suggests learning-induced plasticity in VCN (Figure 5).

## Discussion

We have discovered the presence of neurons in CN that show plasticity to alternating frequencies of tone paired with shock. The change in the rate of neural firing was dissociated in the two subnuclei of the CN. Specifically, the DCN showed a significant decrease whereas the VCN showed a significant increase in responding to the CS paired with shock. DCN change is likely in response to shocks broadly rather than learning-induced, because the decrease in firing at conditioning compared with baseline was observed at both Pre-CS and CS periods. In contrast, the increased excitability of VCN specifically for highly distinctive CS+ and not during pre-CS or less distinctive CS+ suggests that learning-induced plasticity occurs in VCN (Figure 5). This finding is consistent with stronger and long-lasting bradycardia (1.3–8 s from CS onset) observed with heart rate when the CS+ was more distinctive, involving tones more than 3 octaves apart (Figure 4A). With less distinctive CS+ (alternating tones 3 or fewer octaves apart), heart-rate CR showed a pattern of bradycardia (2.7–2.9 s from CS onset) then tachycardia (6.5–8.0 s from CS onset) compared CS− trials. Bradycardia followed by tachycardia has been observed previously as a ‘fear’ response [39], whereas prolonged bradycardia is believed to indicate ‘anxiety’ [40,41]. Dominant theories of fear vs anxiety state that fear is prompted by imminent and real danger to facilitate active defence, whereas anxiety is elicited by less specific/predictable threats that promotes arousal and vigilance to prepare for distal and potential threat. Taken together, the present heart rate results indicate that highly distinctive CS that is paired with shock leads to anxiety, which may suggest vigilance and arousal. Therefore, increased excitability of VCN only for highly distinctive CS+ may contribute toward vigilance and arousal associated with anxiety invoked by CS+ trials compared with CS− trials.

Neural gain in VCN allowing for an enhanced responsiveness to a broader range of frequencies coupled with decreased DCN activity is consistent with the postulated role of DCN inhibition in neural gain control [42]. The relevance of the DCN–VCN connection in this study pertains to possible associative learning induced changes in the balance of inhibition and excitation that exists between these structures for regulating the excitability of neurons. The DCN may play an important role in frequency discrimination [43], which, when reduced, results in significantly broader tuning characteristics and lower thresholds in the VCN [44]. More importantly, the firing regularity of output VCN stellate cells, important for frequency coding, would be directly influenced by this decreased threshold resulting from a decrease in DCN inhibition [42]. Given stellate neurons show greatest firing regularity (termed as chopping) at their characteristic frequency, responding to the changes in frequency feature at CS+ onset may require the integration of this information by broadly tuned neurons upstream. Thus, for frequency discrimination, reducing DCN activity and maximizing VCN output would be advantageous allowing chopping response to be more easily detected.

It is unclear why DCN changes were not selective while VCN changes were selective to the CS+. These findings may relate to CN’s role in auditory perception for higher order processing from the amygdala. The amygdala contains neurons that are broadly tuned and tone-responsive for much longer durations than the stimulus itself [45], suggestive of a process that allows integration of temporally disparate stimuli necessary to facilitate associative learning. The broader the frequency response the greater the probability of integration of disparate frequencies presented over time. When octave differences within the CS+ are large, changes at the level of the brainstem may be crucial to ensure the broadly tuned neurons in the amygdala can process convergent frequency streams to maintain a CR to the temporally disparate tones. Consistent with this proposition is the intriguing observation that the balance of inhibition and excitation appears to be altered depending on the octave difference between stimuli. The greater the octave difference in the CS+ the larger the disparity between VCN and DCN activity was observed, with excitability increasing markedly in the VCN. These results imply a dynamic mechanism requiring increases in the level of gain until detection is reached. The greater the octave difference the broader the required VCN frequency response, as shown by increasing response to the non-characteristic frequency tone toward the end of CS+ trial (Figure 5). This complexity suggests the involvement of both the thalamic-amygdala and thalamic-cortico pathways.

Given the projections of the thalamo-amygdala pathway originate in regions which are not tonotopically arranged [24,46], and many neurons of these regions show broad tuning properties [47], the thalamo-amygdala pathway is unlikely to be sufficient for conditioned fear if fine discrimination is required, as in the present conditioning paradigm involving mismatched tones of varying frequencies. Such a task may require multiple parallel streams. Top-down auditory cortical activation of the auditory midbrain inferior colliculus and subsequent avenues for regulation of the CN via periolivary nuclei [31,48–51] may result in setting the appropriate neural gain necessary for the detection of frequency change. The greater the octave difference the greater the cortical load and ramping of neural gain in the VCN.

Interestingly, the result of VCN discrimination may manifest functionally with only bradycardia at large octave differences to tachycardia/bradycardia at small octave differences. Such differences were not observed during pre-CS periods, indicating that these heart-rate changes are due to conditioning to the CS+. These heart-rate patterns may reflect the degree of certainty or uncertainty regarding whether the frequency pattern is changing, which is biologically significant to determine. High octave differences may mean the shock is certain, hence the rat may revert to passive coping or ‘learned helplessness’, and parasympathetic drive predominates. This is in contrast with uncertainty were the rat attempts to cope or strategize, resulting in increased sympathetic drive [52]. Conditioned fear may drive the attentional processors top-down through the MGN to the VCN to initiate changes in the balance of inhibition and excitation, allowing the CS to be more easily discriminated.

There are a few limitations to note. Heart-rate analyses involved six animals, and electrophysiology analyses involved four animals, which may be considered is low sample size. However, the within-subjects design with hundreds of measurements (e.g., 20 pips in a CS trial) meant that the study was well-powered to detect small to moderate effects at >0.8. Also, movement data such as freezing is not available in the present study. Freezing, defined as absence of movement other than that required for breathing, is the most widely used measure for conditioned reflexes in rodents [53,54]. Although freezing measures provide benefits such as the wide availability of comparison studies, of the three historically established measures of conditioned fear [3], which is the best measure is yet equivocal. For example, it has been argued that heart-rate measurements can capture not only the acute fear response but also more prolonged anxiety-like responses [40] that may be resistant to extinction [39]. Further, heart-rate findings are more readily translatable to human findings [40,55].

In addition, it is unclear whether the behavioral and neural plasticity observed are long-lasting, with the lack of long-term tone-only test following conditioning. Considering that VCN changes did not occur to pre-CS periods suggest that it is likely that VCN changes are due to ‘learning’ that occurred to the CS+. However, whether they reflect CS+ ‘memory’ needs to be determined using tests without any shock. DCN findings appear to represent broad state-dependent modulation, not selective to CS+. The present findings have important implications on our understanding of perception, that processing of sensory information may be dynamically mediated by experience.

## Open Access

Open access for this article was enabled by the participation of University of Melbourne in an all-inclusive *Read & Publish* pilot with Portland Press and the Biochemical Society under a transformative agreement with CAUL.

## Abbreviations

ANOVA: analysis of variance
CN: cochlear nucleus
CR: conditioned response
CS: conditioned stimulus
CT: computed tomography
DCN: dorsal CN
FWER: family-wise error rate
i.p.: intraperitoneal
LA: lateral amygdala
MGN: medial geniculate nucleus of the thalamus
PBS: phosphate buffered saline
VCN: ventral CN
